# Developing biobanks in developing countries

**Published:** 2011-06

**Authors:** Igor Rudan, Ana Marušić, Harry Campbell

## Abstract

We call for the development of human biobanks in developing countries and describe several examples from low income countries which are already building their own biobanks. Developing human biobanks in developing countries requires strengthening of the research capacity to use the new technologies, as well as a shift in research investment priorities in order to reduce the inequity in international research that currently exists. A responsible approach from low-income countries to ethical issues will be another pre-requisite to the success of the ‘hypothesis-free’ research that will target the needs of the world’s poor.

The sequencing of the human genome, completed and reported a decade ago, increased the potential of what could be described as a ‘data-driven’ or ‘hypothesis-free’ approach to biomedical research (1,2). The rise of powerful new technologies for high-throughput analysis of human genetic material resulted in an avalanche of genome-wide association studies (GWAS), which currently contribute to an unprecedented progress in assigning ‘biomedical‘ functions to human genes (3). With massively increased capacity for studying human genetic material, which grew by several orders of magnitude over the past decade, while falling dramatically in price (4,5), we can now measure human genetic make-up more precisely than other human traits or environmental exposures that were traditionally studied in biomedical research – such as dietary habits, blood pressure or biochemical studies of the levels of proteins in the blood (4). Thus far, companies such as Illumina and Affymetrix have managed to provide tools in a form of chip-based technology, which are helping to understand common human genetic variation. Soon it will be possible to sequence the entire human genome, letter-by-letter, at a price under US$ 5000 (€ 3575), and the key limit to further biomedical discoveries may be imposed by the limited capacity of contemporary computers to handle this massive amount of information (5).

In addition to this progress, great advances were made in the high-throughput analysis of so-called ‘-omics’ traits – thousands of circulating molecules and metabolites that were jointly named the ‘metabolome’, ‘proteome’, ‘lipidome’, or ‘glycome’ (6-8). An explosion of information that can now be collected and analysed for each individual led to the development of large ‘human biobanks’, the largest of which are catalogued by the Public Population Project in Genomics (or P3G) (9). These are repositories of human DNA material and plasma samples collected from large number of individuals and stored anonymously along with other information on their lifestyle, diet, anthropometric and physiological measurements, genealogies and psychological well-being. These biobanks all have several things in common. First, they share the principle of adherence to rigorous ethical principles for recruiting participants and for using and handling the collected information. Second, they are very large and store information on many (tens of) thousands of individuals. Third, they provide researchers with an opportunity to maximise the research and clinical and public health translation potential from the new high-throughput research technologies, which require such biobanks to generate important new health knowledge.

Biomedical research that relies on the application of high-throughput technologies in human biobanks can be described as ‘data driven’, ‘hypothesis-free’ science. Traditionally, the advancement of science relied on the accumulated, existing knowledge, which was then used by the researchers to generate and test further hypotheses, thus advancing their field further. This alternate paradigm of biomedical research that relies on human biobanks is not dependent on ‘a priori’ hypotheses, because it can simply apply rigorous statistical methods to search for apparent associations between thousands or millions of variables that were measured simultaneously in a very large sample of participants, using exceptionally precise (and increasingly inexpensive) measurement tools, while correcting for and discarding false-positive results expected due to multiple testing (10).

The diseases that are currently being studied by the wealthy nations would have been almost entirely ‘invisible’ to selection pressures. This may be one of the reasons why the results of genome-wide association studies have not yet found strong genetic effects that could be easily translatable into clinical practice and commercialized.

What is so appealing about this ‘hypothesis-free science’? First, it is virtually free of human bias, opinion, or imbalanced interpretation. It is typically based on extremely accurate measurements, often using very large sample sizes generated through international collaboration of many centres that applied the same measurement methods and a common analysis plan, so that the results are directly comparable, relatively free from bias and confounding and not subject to sampling variations due to small sample sizes. Many recent successes of this ‘hypothesis free’ approach have also exposed that the science of the 20th century – where many small research groups were working in isolation from each other, using small sample sizes and publishing their results independently of each other – was much more likely to report false-positive results (11). It was quite an embarrassment to the field to realize that the vast majority (more than 95%) of the reported results on associations between genes and human traits and diseases in the period before 2007, when the rise of genome-wide association studies begun, were not replicable in much larger and better designed studies (11). Second, and equally importantly – ‘hypothesis-free’ studies do not depend on previous knowledge, thus allowing large leaps forward in scientific discovery, and unexpected and exciting new breakthroughs in understanding (12). However, there is still an important place for hypothesis-driven experiments in following up these findings to understand their full significance in terms of improved knowledge of underlying pathophysiological mechanisms or their health impact if translated into clinical guidelines or public health action.

However, the current state of ‘hypothesis free’ science that relies on human biobanks is not free from concern. A quick look at the biobanks listed in the P3G observatory shows that nearly all of them have been developed to address the health problems relevant to the minority of people living in wealthy countries, mostly the complex chronic noncommunicable diseases of late onset. This reflects the disease burden in these countries and also the potential for research commercialization to address these problems. Ten years after the human genome has been sequenced there are still hardly any biobanks in low and middle income countries. Even among those that exist, only a few seem to address the problems of the poor, which contribute to the majority of global burden of disease. A recent study showed that that nearly all the progress made by the powerful new high-throughput research technologies was currently confined to wealthy countries and their health needs (13).

The human genome has been shaped through continuing struggle of humanity to survive among many other species and in challenging environmental conditions. The strongest effect of human genes should therefore be expected to ensure successful conception and intrauterine growth and development, safe and full-term delivery and resistance to infectious diseases of childhood and early adulthood. These were historically the main selective pressures that could significantly shape the human genome. They are also still the main contributors to the burden of disease in many developing countries today, but they have not yet been the main focus of interest of human biobanks. The diseases that are currently being studied by the wealthy nations would have been almost entirely ‘invisible’ to selection pressures. This may be one of the reasons why the results of genome-wide association studies have not yet found strong genetic effects that could be easily translatable into clinical practice and commercialized (14). Because of this, some opinion leaders are beginning to question this approach (14,15).

We believe that research into health problems of low income countries and the poor may be a better placed endeavour for human biobanks, and it may result in more obvious successes.

We believe that research into health problems of low income countries and the poor may be a better placed endeavour for human biobanks, and it may result in more obvious successes. Some experts have already proposed this, too, based on other considerations, such as needs, feasibility and equity (16-18). We call for the development of human biobanks in developing countries, and praise several examples from low income countries which are already building their own biobanks (19,20). However, this will require strengthening of the research capacity in many low-income countries to enable them to use the new technologies. It will also require a shift in research investment priorities in order to reduce the inequity in international research that currently exists. Finally, a responsible approach from low-income countries to ethical issues will be another pre-requisite to the success of the ‘hypothesis-free’ research that will target the needs of the world’s poor. We explore this topic in the viewpoint (21) and two research articles in this issue of the Journal (22,23).
